# The Impact of a Huge Fibroid on Pregnancy: A Case Report

**DOI:** 10.7759/cureus.71688

**Published:** 2024-10-17

**Authors:** Jasbir Chhatwal, Benish Khanzada, Areesha Kamran

**Affiliations:** 1 Obstetrics and Gynecology, Zulekha Hospital, Dubai, ARE; 2 Obstetrics and Gynecology, Watim Medical and Dental College, Islamabad, PAK

**Keywords:** obstetric outcome, pregnancy, transverse lie, ultrasound, uterine fibroid

## Abstract

Uterine fibroids are benign smooth muscle tumors of the uterus, are relatively common in reproductive-age women, and are associated with increasing maternal age. This case study details a 37-year-old female patient who was incidentally diagnosed with a larger lower segment fibroid during her first pregnancy scan and responded well to treatment, delivered by a cesarian due to transverse lie and lower uterine segment fibroid completely obstructing the birth canal.

## Introduction

A benign tumor of the uterine smooth muscle, fibroids are more common in women who wait until later in life to become pregnant, with a prevalence of 0.1-10.7% during pregnancy [1-3]. Fibroids can affect a woman's daily activities if they become symptomatic during pregnancy and can also impact her fertility and obstetric outcomes. Miscarriages, inadequate fetal growth (IUGR), pain from fibroid degeneration, fetus malpresentation, premature labor, cesarean section, infection, obstetric hysterectomy, antepartum, and postpartum hemorrhage have all been associated with fibroids in pregnancy [4-6].

Women over 30 years of age who become pregnant for the first time have a higher chance of developing fibroids during pregnancy compared to multiparous women. While most fibroids do not cause complications during pregnancy, they can sometimes lead to severe pain, discomfort, and bleeding. Studies suggest that after delivery, fibroid size decreases significantly, with one study showing a decrease in fibroid size in 72% of women postpartum. During the puerperal period of six months, fibroid volume can decrease by 50% [6,7]. Here, we are presenting a case of a 37-year-old asymptomatic woman who presented with a complaint of five weeks of amenorrhea and lower abdominal pain. Ultrasonography revealed a huge intramural lower segment uterine fibroid during her pregnancy.

## Case presentation

**Figure 1:**
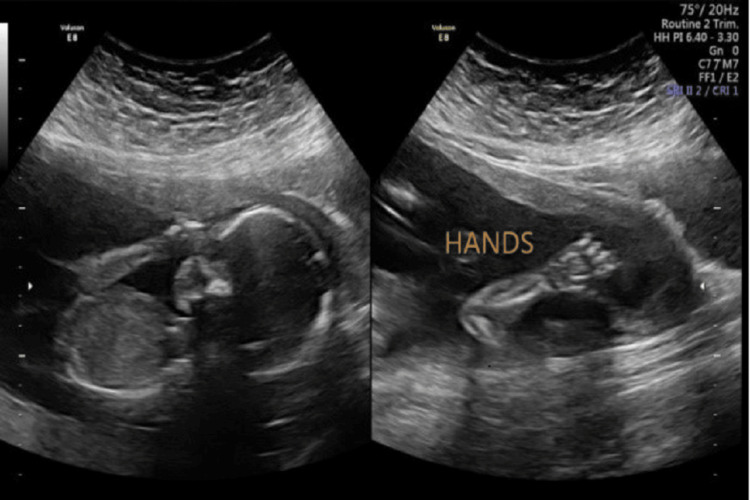
Ultrasound fetus on 11 weaker NT scan NT: nuchal translucency

**Figure 2:**
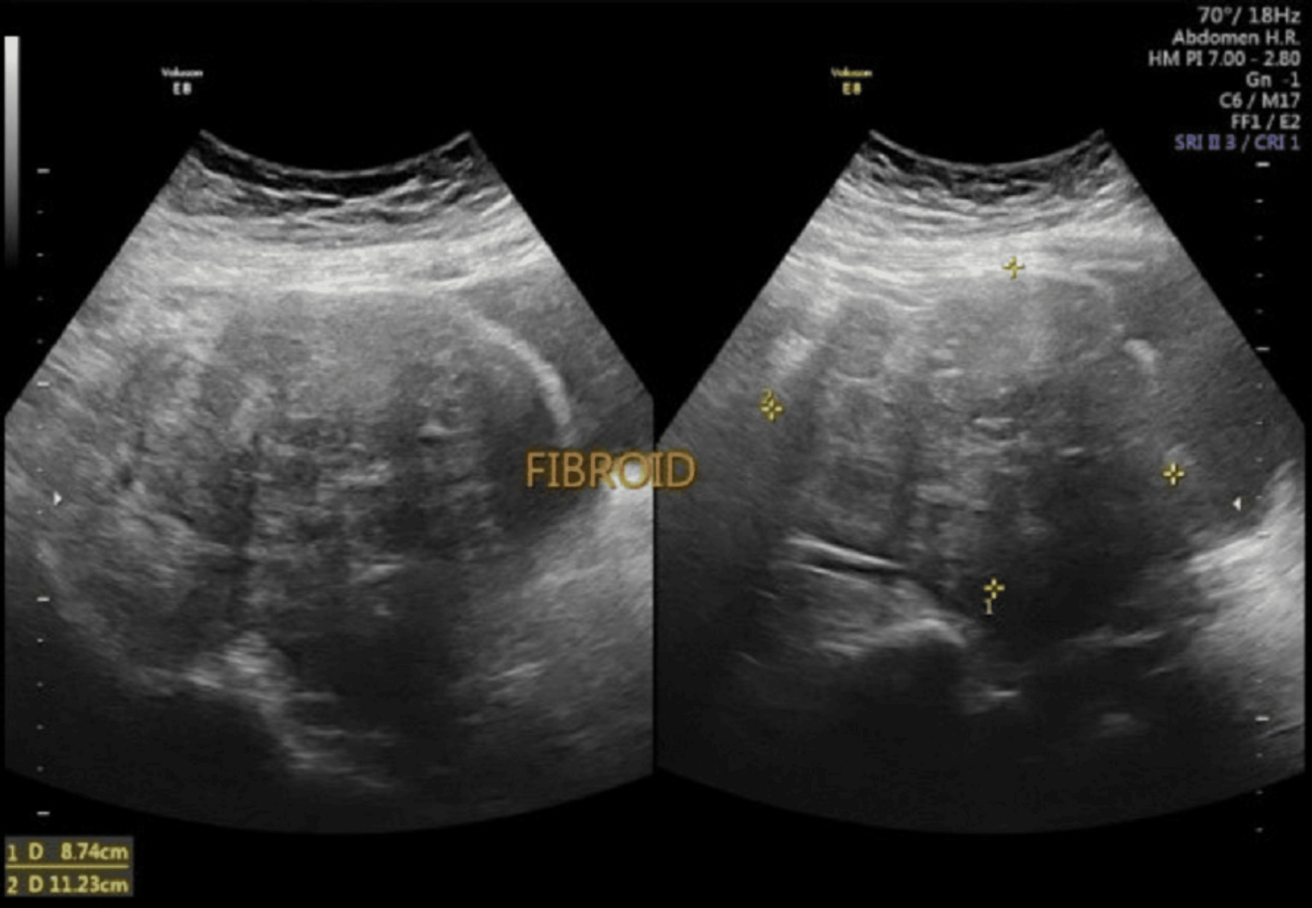
Ultrasound of 11.7 cm lower segment fibroid at 11 weeks of pregnancy

A 37-year-old, elderly primigravida, homemaker, from good socioeconomic status, presented in the antenatal clinic at five weeks of amenorrhea and lower abdominal pain. Pregnancy was confirmed by blood pregnancy test and folate supplementation commenced. A single intrauterine pregnancy was confirmed on ultrasound along with the incidental finding of a large heterogenous abdominal mass of 11.23 × 8.7 cm attached to the uterus (Figures 1, 2).

Cancer antigen 125 (CA 125) and lactate dehydrogenase (LDH) performed to rule out malignancy were normal. At 12 weeks, a nuchal translucency scan was performed and found to be normal with the same size of intramural fibroid occupying the lower uterine segment and cervix. From the start of pregnancy, the patient complained of lower abdominal pain and kept on progesterone support for threatened miscarriage and preterm labor prevention. At 20 weeks, an anomaly scan was done with an evaluation of the fibroid; no changes were noted in the size of the fibroid and normal fetal scan.

The patient was recommended to have routine ultrasound follow-ups to track the fibroid's growth and the development of the fetus. She was prescribed acetaminophen for pain relief, with instructions to avoid nonsteroidal anti-inflammatory drugs (NSAIDs) due to their potential adverse effects on the fetus. Standard prenatal care was provided, with additional monitoring for signs of preterm labor and placental abruption. At 24 weeks diagnosed with gestational diabetes on oral glucose tolerance (OGTT) that was later controlled on diet modification and oral hypoglycemic. Growth scans were done every four weeks until 32 weeks, and 36 weeks verified that a transverse lie and elective cesarean was scheduled for 38 weeks due to the transverse lie and lower segment fibroid filling the pelvis and cervix. The couple was counseled about possible maternal and fetal complications associated with transverse lie and large lower segment intrauterine fibroid, i.e., the need for classical scar, possible interventions, intraoperative blood loss, postpartum hemorrhage, and blood transfusion if needed.

**Figure 3:**
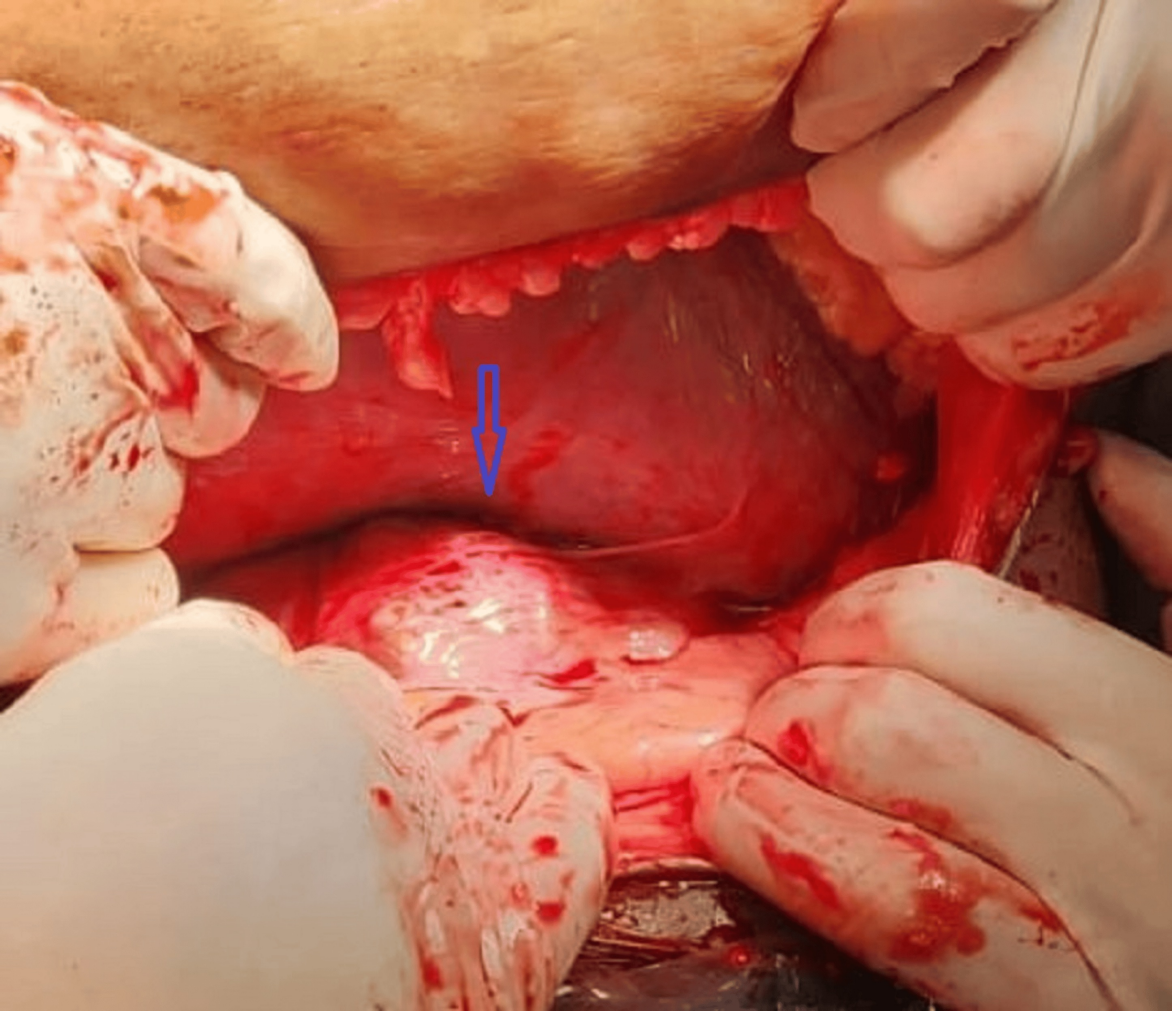
Intraoperative fibroid in lower uterine segment

**Figure 4:**
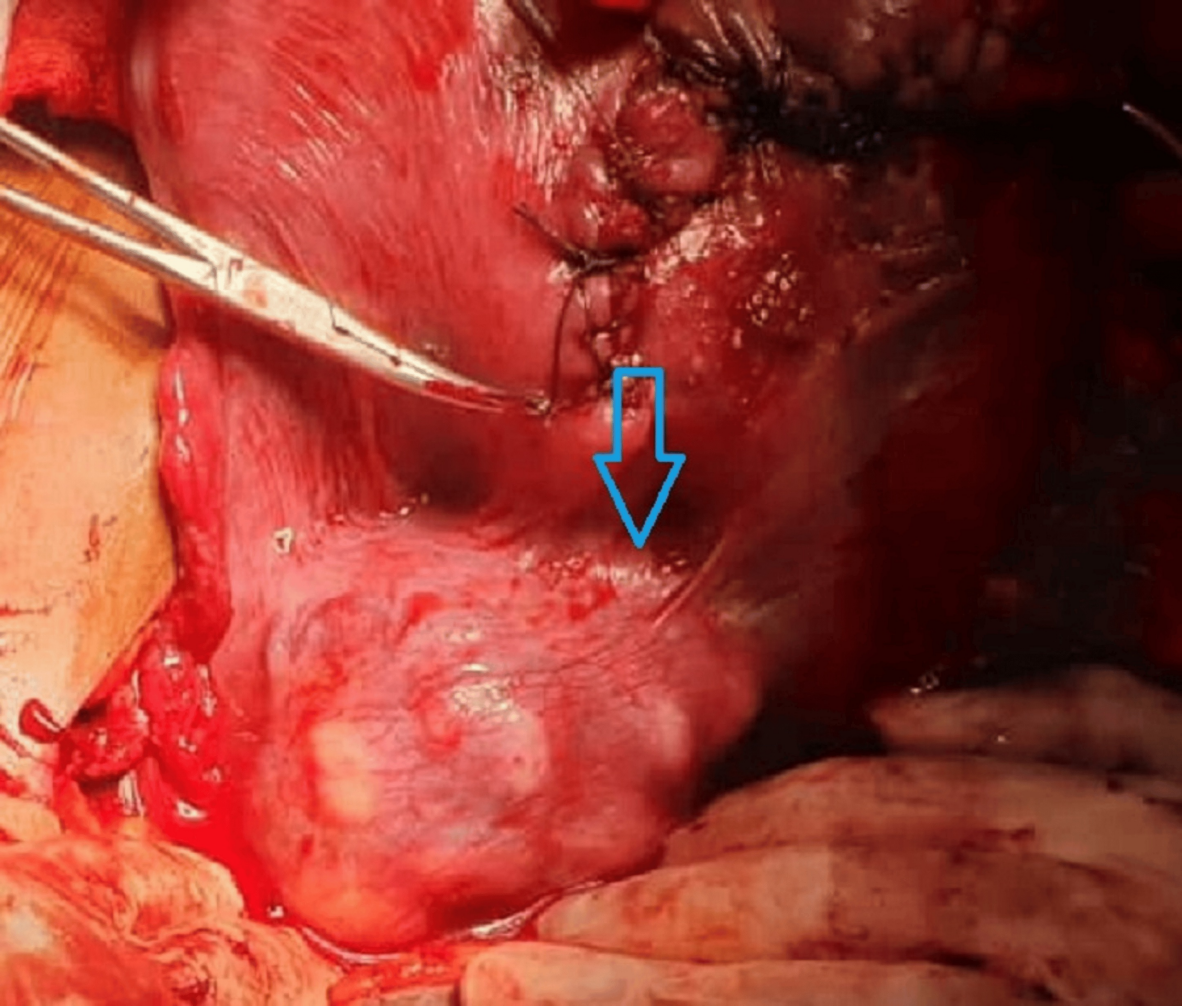
Intraoperative fibroid in lower uterine segment

A possible hysterectomy was also explained and consented to in case of excessive intraoperative or postpartum hemorrhage. A cesarian section was performed under spinal anesthesia, and a transverse incision was given above the fibroid, occupying the lower segment of the uterus anteriorly, and the baby was delivered by breech. The intramural fibroid occupying the lower uterine segment was noted during the C-section (Figures 3-4) but was not removed due to the high risk of hemorrhage.

**Figure 5:**
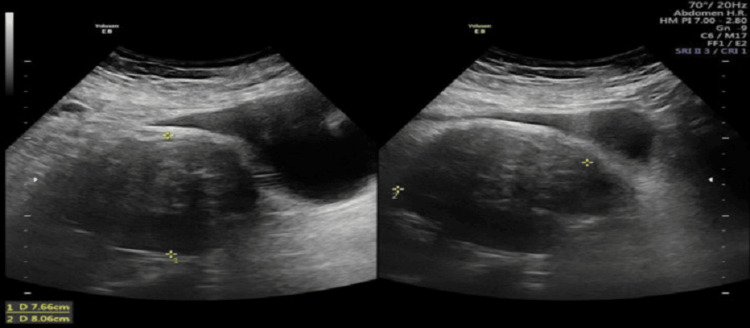
Ultrasound 25 days post-delivery involuted the uterus and decreased the size of the fibroid to 7.6 cm

She delivered a live baby with a birth weight of 3000 grams and with a good appearance, pulse, grimace, activity, and respiration (APGAR) score. The placenta was removed completely without excessive intra- or postoperative hemorrhage. On the third postoperative day, she was released from the hospital, and her postoperative recovery was uneventful. The patient complained of pain on the 25th postnatal day. An ultrasound of the abdomen was recommended, and the results revealed a striking reduction in size to 7.6 cm × 8.6 cm in the lower uterine section that extended into the cervix (Figure 5).

At the six-week postpartum follow-up, the patient was asymptomatic, and the uterus had been involuted appropriately.

## Discussion

Uterine fibroids are associated with an increased risk of early and late pregnancy losses, especially if they are in the upper uterine body than the lower uterine segment [7-9]. Uterine irritability and contractility due to abnormal fibroid musculature compressive effect of uterine fibroid and disruption of blood flow to the fetus and placenta can lead to miscarriage, intrauterine growth restriction, and preterm labor [10-12]. This effect is more pronounced if the placenta implants close to the fibroid musculature and also depends on the location of the fibroid [13-15].

In our case, the patient complained throughout pregnancy of lower abdominal pain, and we kept her on progesterone as a prophylactic measure to prevent miscarriage and preterm labor. Based on cohort studies, pregnancy with uterine fibroids can lead to preterm birth (gestational age (GA) <37 weeks) (OR 2.27 (0.30-3.96)) and extreme preterm birth (GA 22-28 weeks, OR 20.09 (8.014-50.22) [16].

Cervical fibroids can enlarge during pregnancy and grow toward the uterus. They can cause obstructed labor, preterm labor, and antepartum hemorrhage, and sometimes abortion may need emergency intervention if such situations occur [17-19]. In a review of randomized control trials by Jenabiet al., there was a statistically significant relation between leiomyoma and cesarian (2.60; 95% CI: 2.02, 3.18) and between uterine fibroid and malpresentation [20]. In our case, fibroid was in the lower uterine segment, completely obstructing the birth canal. As a result, the fetus was in a transverse position and delivered by cesarean. However, normal vaginal delivery is possible in cases where the fibroid is not coming in the way of the fetus by obstructing its way to descend in the birth canal and cephalic presentation. Postpartum size [6,7] decreases post-delivery in 50-70% of women. Our study also favors the same decrease in size from 11 cm to 7.7 cm at 25 weeks post-delivery.

## Conclusions

In conclusion, uterine fibroids during pregnancy are likely to increase in the coming years with increasing maternal age. It increased the risk of malpresentation and cesarian delivery. In this case, the patient was primigravida and was unaware of uterine fibroid, diagnosed incidentally on an early pregnancy scan. It was deemed to be a wise decision to continue pregnancy while keeping an eye on fetal and maternal well-being, as we were able to wait till term.

Making the decision to have a cesarian myomectomy in a rare clinical circumstance might be challenging. The decision depends on the location, surgeons' skill, availability of blood, blood products, and intensive care unit. From our point of view, further case reports and series should be published to share experiences and support the best medical practice.
